# Influence of profilin on sensitisation profiles determined by cutaneous tests and IgE to major allergens in polysensitised patients

**DOI:** 10.1186/s13601-016-0114-y

**Published:** 2016-06-29

**Authors:** Nieves Segura, Teresa Abos, José A. Compaired, Esther Compés, Isabel Guallar, Manuel Morales, Susana Monzón, José Mozota, Pilar Muñoz, Jesús Pola, Macarena Quintana, Beatriz Rojas, Sara San Juan, Felicitas Villa, Cristina Zapata, Lucía Jimeno, Fernando de la Torre

**Affiliations:** Hospital Clínico Universitario, Saragossa, Spain; Consorcio de Salud de Aragón, Huesca, Spain; Private Office, Huesca, Spain; Hospital Royo Villanova, Saragossa, Spain; Hospital Miguel Servet, Saragossa, Spain; Consorcio de Salud de Aragón, Saragossa, Spain; Hospital Militar, Saragossa, Spain; Hospital Obispo Polanco, Teruel, Spain; Policlínica Sagasta, Saragossa, Spain; ALK-Abelló, S.A., C/Miguel Fleta, 19, 28037 Madrid, Spain

**Keywords:** Component-resolved diagnosis, Skin prick test, Profilin, Polysensitisation

## Abstract

**Background:**

Profilin sensitisation is considered a diagnostic confounding factor in areas where patients are exposed to multiple pollens. The aim of this study is to assess pollen sensitisation profiles in adults and children and to evaluate, by means of component-resolved diagnosis (CRD) and skin prick testing (SPT), which pollens may be considered as risk factors of profilin sensitisation in order to establish the best diagnostic approach in polysensitised patients.

**Methods:**

A total of 231 pollen-allergic patients (adults and children) were included, out of the pollen season, from an area with similar levels of pollen exposure. Allergological diagnosis was performed by SPT and determination of specific IgE (sIgE) to major allergen components (ADVIA-Centaur™). Patients had not received immunotherapy in the last 5 years and had to reside in the area for 5 consecutive years before entering the study.

**Results:**

The relation between sensitisation measured by SPT and by sIgE was studied using a model of cases (patients with +sIgE to a specific allergen) and controls (patients with −sIgE to the same allergen). The outcome, in terms of odds-ratios (OR), was statistically significant for Olea (Ole e 1) (p = 0.0005), Salsola (Sal k 1) (p = 0.0118) and Platanus (Pla a 1+ 2) (p = 0.0372). While positivity of SPT to most pollens was statistically associated with a risk of profilin sensitisation, by CRD the association was statistically significant only for Ole e 1 (OR 3.5, CI 95 %, 1.6–7.6, p = 0.0014), and Phl p 5 (OR 11.9, CI 95 %, 4.1–35.2, p < 0.001). When analysing this association using a logistic regression model, Phl p 5 was the only allergen associated with the risk of being sensitised to profilin (p = 0.0023).

**Conclusions:**

In patients sensitised to profilin, the concordance between SPT and CRD is much lower than in those not sensitised to profilin. CRD is able to provide refined information about which pollens increase the risk of sensitisation to profilin.

## Background

Immunoglobulin E (IgE)-mediated allergic diseases, mainly asthma and allergic rhinitis, are highly prevalent diseases, affecting hundreds of millions of people worldwide [1, 2]. The inherent costs of these diseases, especially in developed countries, are extremely high [3, 4]. Therefore, defining an adequate diagnostic strategy is crucial in order to establish the best therapeutic option and consequently, reduce the economic burden of allergic diseases. Until recently, the allergological diagnosis in clinical practice was mainly based on the IgE response against whole allergen extracts, either assessed by means of skin prick test (SPT) and/or by specific IgE (sIgE). However, the sensitisation profile of allergic patients in complex pollen areas reveals that most patients are polysensitised [5]. In these patients, the use of conventional techniques may result insufficient to establish an adequate diagnosis [6]. Recent studies have shown that frequently polysensitisation may be due to cross-reactivity caused by sensitisation to panallergens, such as profilin, polcalcin or lipid-transfer proteins [7, 8]. Sensitisation to these molecules makes it difficult to discern whether a positive test to a whole extract, either by SPT or sIgE, is positive due to a primary sensitisation or a cross-reactivity phenomenon.

Component-resolved diagnosis (CRD) provides a more specific diagnosis in patients with positive IgE to multiple pollen allergens. The main outcome is to learn the sensitisation profile of allergic patients and consequently, to allow a more precise prescription of immunotherapy including only relevant allergens [9, 10].

Some of the aforementioned studies have been performed with patients from different geographical areas and consequently, with different sensitisation profiles as well as different allergen exposure. Therefore, extrapolating conclusions, although valid, may entail certain bias [5, 7]. In our study we have analysed allergic patients from one geographical area, with the aim of (a) establishing the sensitisation profiles in the selected area to the more prevalent aeroallergens by means of SPT and CRD as well as the differences in these profiles according to age, type of allergic respiratory disease and presence of plant-food allergy, (b) studying the differences between both diagnostic techniques in patients sensitised to the main panallergen in the area (profilin) and (c) in profilin-sensitised patients, establishing the risk-factors associated with being sensitised to profilin.

## Methods

### Geographical area of study

**Fig. 1:**
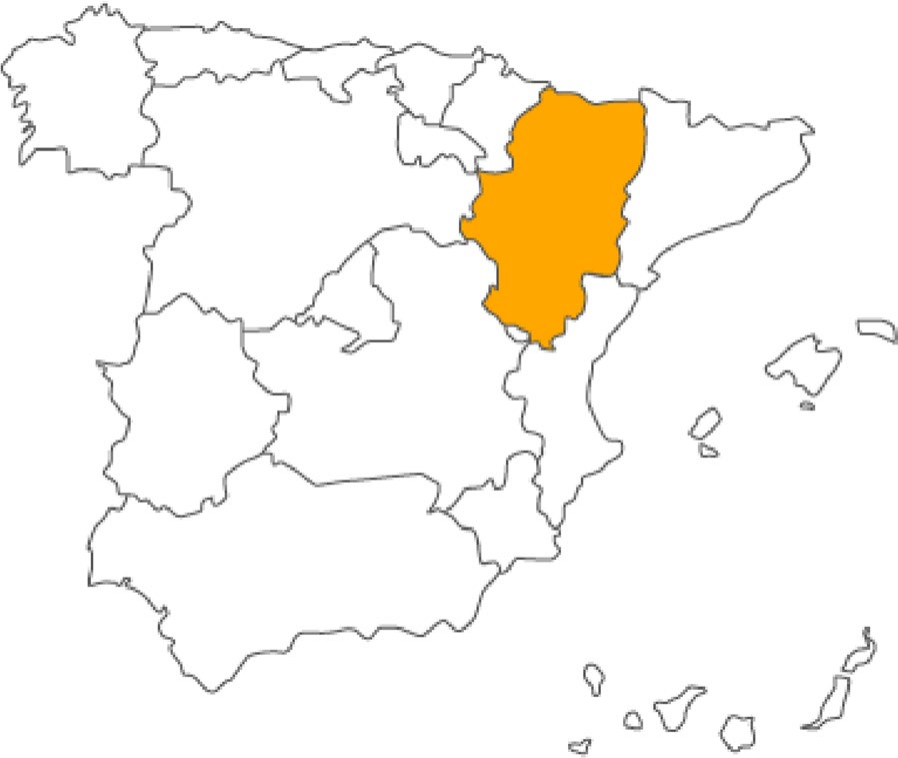
Geographical area of the study

The study has been carried out in a geographical area (Aragon, Spain) with a continental Mediterranean climate with cold winters, dry and hot summers and with areas of high mountains (see Fig. 1).

### Patients

Patients included in the study were selected consecutively for two periods in the same year before and after the pollen season. All patients had a clinical history of seasonal allergic rhinitis, rhinoconjunctivitis or asthma for at least 2 years, should not have received immunotherapy in the last 5 years, according to the same methodology used in previous studies [5, 7] and had to reside in the area of the study for 5 consecutive years or longer before being included in the study.

Clinical diagnosis of allergic rhinitis and asthma was performed according to international and national guidelines [11, 12].

Written informed consent was obtained from each patient before entering the study. In case of patients aged <18 years, informed consent of parents/guardians was also obtained. The study was approved by the regional ethical committee (Comité Etico de Investigación Clínica de Aragón).

Serum samples were collected from the subjects, stored at −40 °C and thawed immediately before analysis.

### Skin prick test extracts

Natural profilin, Pho d 2 extract, was prepared by purifying a date palm extract by affinity chromatography with a poly-l-proline-Sepharose. Purity (higher than 99 %) was checked by SDS-PAGE, mass spectrometry and amino acid analysis. The concentration of Pho d 2 in the extract was 50 µg/ml. Date palm polcalcin enriched extract was obtained from the same extract (showing total protein concentration of 500 µg/ml after Lowry) after removal of profilin. In a previous experiment, positive SPT responses to complete Palm tree extracts were assigned either to profilin or polcalcin sensitised patients, but not to LTPs, CCDs or Glucanases. Protein identity was assessed by SDS-PAGE. The concentration of polcalcin, measured by an inhibition assay against r-Che a 3, was determined to be 1 µg/ml.

A commercial peach extract from ALK-Abello S.A., adjusted to 30 µg/ml of Pru p 3, was shown to lack other relevant allergens (such as Pru p 1 and Pru p 4).

The other diagnostic extracts used in the study (*Olea*, grass-mix, *Artemisia*, *Salsola*, *Cupressus, Parietaria, Platanus* and *Plantago*) were complete commercial extracts from ALK-Abello S.A. at 30 HEP/ml. Alternaria extract is standardised in μg/mL of the major allergen Alt a 1 (25 μg/mL). These pollens were selected because they are the most prevalent sensitizing allergens in the area.

### Panel of purified allergens for sIgE determination to major allergens

The panel of allergens included in the study were: nPhl p 1 and nPhl p 5, nOle e 1, nArt v 1, nCup s 1, nPar j 2, nPla a 1+ 2, nPla l 1, nSal k 1. The panallergens studied were: nPho d 2 (profilin), rChe a 3 (polcalcin) and rPru p 3 (LTP). sIgE against Alt a 1 was also determined due to the clinical relevance of this allergen in the area, not studied in previous research [7] and also because the exposure to grass and Alternaria may be linked to severe asthma [24].

The manufacturing process of all these allergens has been previously described [5, 7, 13].

Specific IgE to the different allergens was tested with the ADVIA Centaur^®^ platform (Bayer HealthCare Diagnostics Division, Tarrytown, NY, USA). The principle of the sIgE assay is based upon a reverse sandwich assay and was performed according to previously established methods [14].

Specific IgE was considered positive when ≥0.35 kU/l.

### Statistical methods

In order to analyse qualitative variable association, Pearson’s Chi square test was used when variables fitted all required assumptions and Fisher’s exact test when not. A multivariate analysis by means of logistical regression by determination of the odds ratio was used to evaluate the level of risk of being sensitised to profilin.

## Results

### Sample description

A total of 231 patients were included in the study. Patients characteristics are described in Table 1. The mean (SD) age of patients was 22.6 (13.6) years, median (range): 18 (4–65) years. Despite food allergy not being an inclusion criterion, almost 20 % of patients had allergic food reactions.

**Table 1:** Characteristics of patients

	%
Age	
≤14 years	45.9
>14 years	54.1
Sex	
Male	49.1
Female	50.9
Clinical diagnosis	
Rhinitis	98.7
Conjunctivitis	81.8
Asthma	46.8
Mild intermittent	63.0
Mild persistent	25.9
Moderate	11.1
Food allergy	18.7
Oral allergy syndrome (OAS)	13.4
Fruits	5.2
Nut	3.5
Fruits and nut	3.0
Seafood	1.3
Urticaria/angioedema	7.8
Fruits	3.0
Nut	2.6
Anaphylaxis	1.3
Asthma	0.4

### Sensitisation profiles

**Fig. 2:**
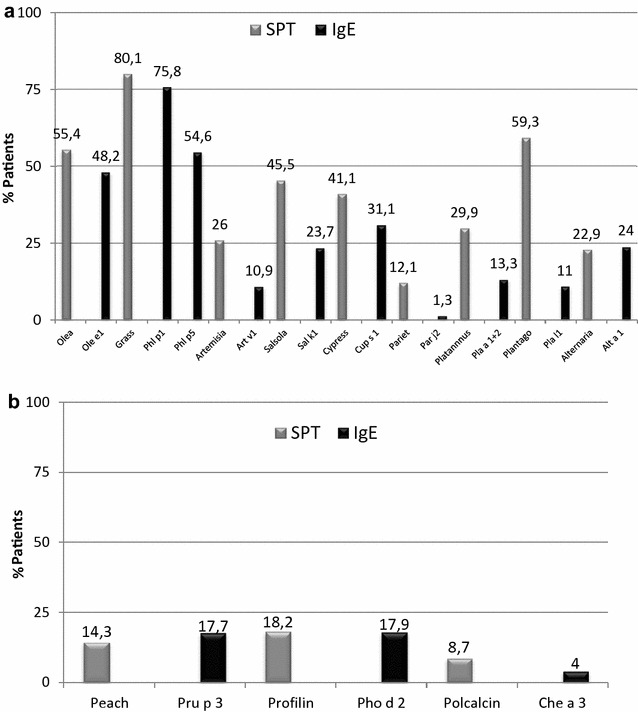
Prevalence of major allergens measured by SPT and sIgE, **a** Pollens and Alternaria, **b** panallergens

In Fig. 2a, b we can see the sensitisation rates by SPT and sIgE to major allergens. The statistically significant differences on the sensitisation profile according to demographic and clinical parameters studied are shown in Table 2.

**Table 2:** Differences on sensitisation profile according to demographic and clinical parameters

	Age		*p* value
	≤14 years	>14 years	
SPT+			
*Alternaria*	32.1	15.2	0.0024
*Cupressus*	32.1	48.8	0.0100
Polcalcin	16	25.6	0.0498
sIgE+			
Alt a 1	35	14.8	0.0004
Cup s 1	19.4	40.7	0.0006

	Sex		
	Male	Female	
SPT+			
Peach	35	14.8	0.0034
sIgE+			
Pru p 3	60.9	9.1	<0.0001

	Asthma		
	Yes	No	
sIgE+			
Sal k 1	16.2	30.1	0.0139

	Food allergy: OAS		
	Yes	No	
SPT+			
*Artemisia*	58.1	21	<0.0001
*Parietaria*	26.8	10	0.0121
*Platanus*	51.6	26.5	0.0045
Peach extract	45.2	9.5	<0.0001
Profilin	32.3	16	0.0290
sIgE+			
Phl p 5	64.5	45.4	0.0478
Pru p 3	54.8	11.8	<0.0001

	Food allergy: systemic reactions (no OAS)		
	Yes	No	
SPT+			
*Artemisia*	58.1	23.6	0.0118
Peach extract	61.3	9.1	<0.0001
sIgE+			
Pru p 3	69.6	11.8	<0.0001

The figures correspond to percentage of patients with a positive test (SPT or sIgE). Only statistical significant results are displayed

*OAS* oral allergy syndrome

The percentage of patients sensitised to Alternaria was 23 % by SPT and 24 % by sIgE to its major allergen Alt a 1.

### Comparison of results in patients sensitised to profilin

Patients sensitised to profilin were sensitised to a higher number of allergen sources, when compared to profilin-negative patients; this was true, both for SPT (mean of 9 allergens vs. 5 allergens, p < 0.0001) and sIgE (mean of 4 allergens vs. 2 allergens, p < 0.0001). In both groups, the relation between sensitization measured by SPT and by sIgE was studied by means of a study of cases (patients with positive sIgE to a specific allergen) and controls (patients with negative sIgE to the same allergen). The outcome, in terms of odds-ratios (OR), was statistically significant for *Olea* (Ole e 1), *Salsola* (Sal k 1) and *Platanus* (Pla a 1+ 2) (Table 3) indicating that concordance between both diagnostic techniques is much higher for patients not sensitised to profilin.

**Table 3:** Relation between allergens measured by SPT and allergens measured by sIgE in patients with positive sIgE to Pho d 2 (profilin+) versus negative sIgE to Pho d 2 (profilin−)

	Allergen								OR	CI 95 %	p value
	Ole e 1+				Ole e 1−						
	SPT *Olea*+		SPT *Olea*−		SPT *Olea*+		SPT *Olea*−				
	n	%	n	%	n	%	n	%			
Pho d 2+	24	88.9	8	66.7	3	11.1	4	33.3	4.0	21.8–0.73	0.0005
Pho d 2−	66	97.1	22	19.5	2	2.9	91	80.5	136.5	600.7–31.0	

	Sal k 1+				Sal k 1−						
	SPT Sal+		SPT Sal−		SPT Sal+		SPT Sal−				
	n	%	n	%	n	%	n	%			
Pho d 2+	11	84.6	17	63.0	2	15.4	10	37.0	3.2	17.7–0.6	0.0118
Pho d 2−	38	92.7	35	24.5	3	7.3	108	75.5	39.1	134.5–11.4	

	Pla a 1+ 2+				Pla a 1+ 2−						
	SPT Pla+		SPT Pla−		SPT Pla+		SPT Pla−				
	n	%	n	%	n	%	n	%			
Pho d 2+	3	60	26	74.3	2	40	9	25.7	0.5	3.6–0.07	0.0372
Pho d 2−	11	45.8	26	16.4	13	54.2	133	83.6	4.3	10.7–1.8	

*OR* odds-ratio, *CI* confidence interval. Sal: *Salsola*. Pla: *Platanus*

p value: Breslow–Day test for OR homogeneity

The risk of being sensitised to profilin was assessed for SPT and sIgE results. For SPT, different pollens are statistically associated with the risk of being sensitised to this panallergen (*Olea* p = 0.04, Grass p = 0.0006, *Artemisia* p = 0.0135, *Salsola* p = 0.0044, *Platanus* p < 0.0001 and *Plantago* p < 0.0001). However, when this risk is analysed considering the sIgE (>0.35 kU/l) despite SPT, the only allergens associated with risk are Ole e 1 (OR 3.5, CI 95 %, 1.6–7.6, p = 0.0014), and Phl p 5 (OR 11.9, CI 95 %, 4.1–35.2, p < 0001). Applying a logistic regression model, the only allergen with a statistically significant risk is Phl p 5 (p = 0.0023). Analysing this OR for different levels of sIgE to Phl p 5, the risk decreases but remains statistically significant (sIgE > 10 kU/l: OR 7.6, CI 95 %: 3.3–35.2, p < 0001. sIgE > 50 kU/L: OR 5.2, CI 95 %: 2.4–11.3, p < 0001).

### Food allergy

The presence of food allergy is associated with different allergens depending on the clinical reaction. Oral allergy syndrome (OAS) as the only clinical manifestation of food allergy was statistically associated with different allergens by SPT (*Artemisia*, p < 0.0001: *Parietaria*, p = 0.0121: *Platanus*, p = 0.0045, peach, p < 0.0001 and profilin, p = 0.0290) and by sIgE (Phl p 5, p = 0.0478 and Pru p 3, p < 0.0001, being close to statistical significance for Art v 1, p = 0.0661 and Pho d 2, p = 0.0801). However, when the clinical manifestation of food allergy was more severe (urticaria, angioedema, anaphylaxis) the association for SPT results is only significant for *Artemisia* (p = 0.0118) and peach (p < 0.0001) and by sIgE, only for Pru p 3 (p < 0.0001).

## Discussion

Nowadays, to perform a diagnostic workup of allergic diseases based only on conventional techniques such as SPT or sIgE to whole extracts may result insufficient in many patients. Allergic patients frequently present sensitisation to multiple allergens, both children [15] and adults, and it is not always possible to establish which allergens are positive due to genuine sensitization or which allergens are positive due to a phenomenon of cross-reactivity. CRD has been proposed by different authors [9, 16–20] as an essential diagnostic tool, not only for establishing the sensitisation profile of patients but also because it may aid in selecting the most adequate composition of allergen immunotherapy.

Many recent publications have pointed out the role of panallergens as a confusion factor for the correct diagnosis of allergic sensitization [5, 7, 8, 21]. Therefore, the diagnostic algorithm in complex allergen areas must take into account the sensitisation (or not) to these allergens. Several diagnostic algorithms have been proposed [7, 9].

In this study, when we compare the adult and paediatric populations, we see that both conventional diagnostic techniques and CRD perform similarly. Alternaria sensitization is more prevalent in children than in adults, both by SPT and CRD. Previous studies, or studies in which sensitisation to Alternaria is a risk factor in the adult population for severe asthma [23], have shown that humidity and fungi such as Alternaria are associated with a greater sensitisation rate in the infant population aged 5–6 years [22]. In this study, this kind of association was not observed, probably due to the type of patient included.. Only sensitisation to Sal k 1 appears to be associated with a distinct clinical expression, being more prevalent in patients with rhinitis than in those with asthma. Differences were observed between results obtained by SPT and those obtained by CRD in patients with food allergy. Whereas there are different allergens associated with the presence of OAS by SPT, probably due to the presence of lipid-transfer proteins (LTPs) in these extracts, by CRD we see that OAS is associated with sensitisation to two allergens (Phl p 5 and Pru p 3), while severe reactions are associated exclusively with Pru p 3. The model of patients with pollinosis and food allergy is complex, although its study allows us to gain insight to some characteristics of allergic sensitization and clinical reactivity.

The confounding effect of sensitisation to profilin on the interpretation of diagnostic techniques has already been highlighted in previous studies conducted according to a methodology similar to our own [5, 7]. Through a case–control study, we can see that in patients sensitised to the major allergens of *Olea, Salsola* and *Platanus*, the concordance of SPT and CRD is much greater in the absence of profilin sensitization. In the case of sensitisation to Phl p 5, the differences were almost statistically significant (p = 0.067); no significant differences were recorded for the remaining allergens. These results may indicate that profilin sensitization is related with the most prevalent pollens in the area of study, and consequently with higher clinical relevance, excluding Plantago (high prevalence by SPT but low prevalence of sensitization to its major allergen Pla l 1) and Cupressus. The lack of relation between sensitization to cypress-pollen and profilin has been previously observed [25]. When assessing the risk of sensitisation to profilin, by SPT, multiple allergens are associated with this risk (grass, *Olea*, *Salsola*, *Artemisia*, *Platanus* and *Plantago*) and it is difficult to establish which one is the culprit pollen. However, when we determine this risk measuring the sensitisation to major allergens by means of CRD, the association is only statistically significant for the two more prevalent allergens: Ole e 1 and Phl p 5. However, unlike in other studies where there are areas with very high grass allergen concentrations [5], the risk of sensitisation to profilin not only increases in line with IgE values, which, in turn, increase in response to Phl p 5, but the OR value actually decreases. This difference is probably attributable to the fact that grasses are the most prevalent allergen in the area studied, though there are other allergens (*Olea*, *Salsola* and *Cupressus*) that are also relevant where there is no one visibly dominant pollen.

## Conclusion

Panallergen sensitization is a major confounder factor when using conventional diagnostic techniques (whole extract SPT or sIgE). CRD is an essential tool for determining the risk-factors associated with panallergen sensitisation, allowing to overcome these pitfalls.
